# miR-214-3p Deficiency Enhances Caspase-1-Dependent Pyroptosis of Microglia in White Matter Injury

**DOI:** 10.1155/2022/1642896

**Published:** 2022-08-22

**Authors:** Liufang He, Tingyan Wei, Yong Huang, Xueli Zhang, Dongbo Zhu, Huazhen Liu, Zhangxing Wang

**Affiliations:** ^1^Department of Neonatology, Affiliated Longhua People's Hospital, Southern Medical University (Longhua People's Hospital), Shenzhen, Guangdong 518190, China; ^2^Section of Immunology, The Second Affiliated Hospital of Guangzhou University of Chinese Medicine, Guangzhou, Guangdong 510405, China

## Abstract

White matter injury (WMI) is the most frequent impairment of neurodevelopment in preterm infants. Here, we report that the caspase-1 inflammasome is abundantly activated in the microglia of WMI mice and results in increased pyroptosis of microglia. Pharmacology inhibition of caspase-1 cleavage alleviated the pathogenesis of WMI mice. The expression of microRNA miR-214-3p was largely reduced in the microglia of WMI mice compared to controls. Compromised expression of miR-214-3p on microglia gives rise to the inflammasome activation and microglial pyroptosis. Treatment with miR-214-3p agomir is sufficient to relieve the white matter lesion and demyelination in WMI mice. miR-214-3p is able to bind to the 3′ region of the NLRP-3 inflammasome compartment NEK7, preventing the transcription of NEK7 mRNA. As a result, in WMI mice, the lack of miR-214-3p leads to the accumulation of NEK7 which supports NLRP 3 inflammasome activation, microglial pyroptosis, and white matter pathogenesis.

## 1. Introduction

White matter injury (WMI) is one of the major pathological features in preterm born infants [1]. WMI mainly manifests as oligodendrocyte lineage disorder and dysmyelination, which may cause further neurodevelopmental deficiency and neurodysfunction [2]. During brain development, microglia are frequently found surrounding the white matter tracts. As the immune cells reside in the central nerve system, by producing proinflammatory cytokines, microglia are closely related to the progress of WMI [3]. The activation of microglia was proven to be involved in the pathogenesis of neuroinflammation and cognitive function impairment [4, 5]. Through the activation and proliferation of microglia were discovered in the patients and the experimental animal models of WMI [6, 7], the detailed regulation methods and mechanisms remain undisclosed.

Besides cytokine secretion, microglia express a wide range of pattern recognition receptors (PRRs) which allow them to respond to the pathogen-associated molecular patterns (PAMPs) and damage-associated molecular patterns (DAMPs) [8]. Upon sensing PAMPs or DAMPs, the NOD-like receptors containing pyrin domains (NLRPs) composed of inflammasomes are rapidly activated [9]. Canonical inflammasome activation was initialized by the priming of NLRP3; the adaptor protein ASC (apoptosis-associated speck-like protein containing a CARD) was then recruited to the complex. The assembly of the NLRP3 complex triggers the cleavage of procaspase-1 to form the heterodimer p20 and p10. In consequence, matured IL-1*β* and IL-18 are generated and secreted as a result of pro-IL-1*β* and pro-IL-18 cleavage [10]. In recent years, a serine-threonine kinase NIMA-related kinase 7 (NEK7) was reported to be another essential compartment of the NLRP3 complex, which was also required in the process of caspase-1 inflammasome activation [11]. The activation of the inflammasome induces the cell to undergo a special programmed cell death called pyroptosis. NLRP3 activation in the M1 microglia was reported to be related to the injury in the ischemia cerebral stroke [12]. Recently, study suggested that the pyroptosis of microglia appears to be involved in the initiation and pathogenesis of WMI [13].

MicroRNAs (miRNAs) are a group of endogenous small noncoding RNAs. By binding to the 3′-untranslated region (3′-UTR) of targeted messenger RNA (mRNA), the major function of miRNAs is inducing mRNA degradation and preventing protein translation [14]. In the past few decades, a wide range of cellular functional signals and disease progression are reported to be regulated by the miRNAs; recent studies indicate that miRNAs may also be involved in the regulation of inflammasome activation in several diseases [15]. A few miRNAs were reported to participate in the development of WMI [16] as well, but the detailed role of how miRNAs regulate WMI has not been studied yet.

Here, we reported that the expression of miR-214-3p dramatically dropped in the microglia of WMI mice. The reduction of miR-214-3p results in the elevation of caspase-1-dependent pyroptosis of microglia. Mechanistically, miR-214-3p specifically binds to the 3′ end of NEK7 mRNA which leads to the degradation of NEK7 mRNA and ultimately the activation of caspase-1 inflammasome in WMI microglia. Both miR-214-3p recovery and caspase-1 inhibition successfully rescued microglia from pyroptotic cell death and WMI pathogenesis.

## 2. Results

### 2.1. Caspase-1-Dependent Pyroptosis Boosted in Microglia of White Matter Injury Mice

**Figure 1:**
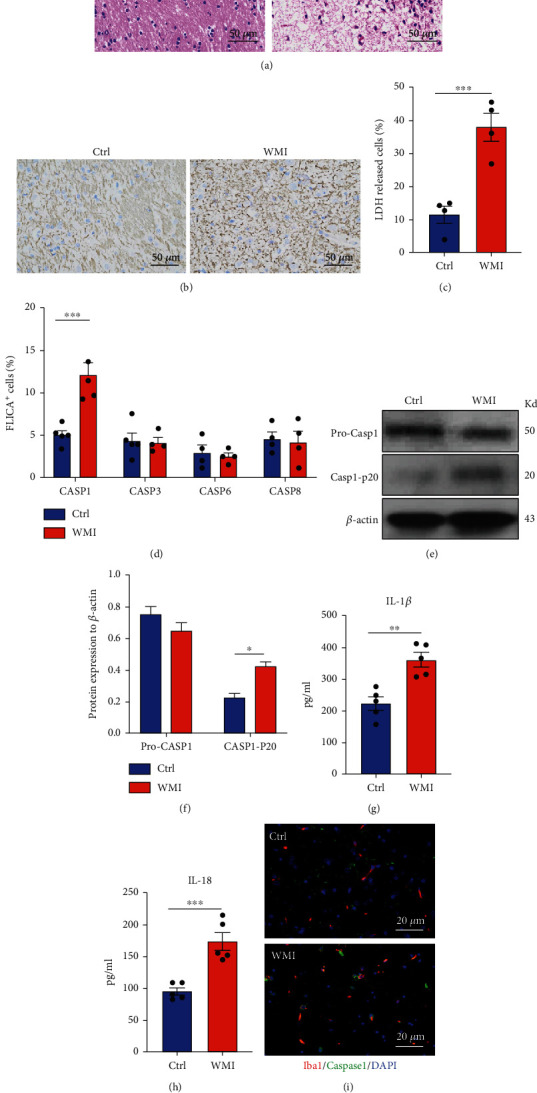
Caspase-1-dependent pyroptosis boosted in microglia of white matter injury mice. 300 *μ*g/kg LPS was injected into mice intraperitoneally on postnatal day 8. 14 hours later, mice were anesthetized, and the brain was harvested. (a) H&E staining for brain sections; white matter was imaged. (b) MBP staining for brain sections. Microglia were isolated with a CD11b cell separation kit. (c) LDH is released by microglia after ex vivo culture for 24 hours. (d) FLICA assay staining in microglia for activated caspase-1, caspase-3, caspase-6, and caspase-8. (e) Immunoblot for microglia from control (Ctrl) and WMI mice. (f) Protein level quantification by normalizing with *β*-actin. (g) IL-1*β* release by microglia. (h) IL-18 release by microglia. (i) Dual-color staining for lba1 and caspase-1 in the white matter. Data are mean ± SEM. Individual data points are displayed: ^∗^*P* < 0.05, ^∗∗^*P* < 0.01, and ^∗∗∗^*P* < 0.001.

Microglia are the major immune cells in the central nervous system (CNS), which patrol in the CNS microenvironment and execute the host defense of the neural parenchyma. To investigate the microglial behavior in white matter injury (WMI), we first established a lipopolysaccharide- (LPS-) induced WMI mouse model as previously described [17]. Compared with the PBS-injected control group, LPS injection leads to an enlarged lesion area in the white matter (Figure 1(a)). Dysmyelination of the white matter tracts is the most featured outcome in WMI; myelin basic protein (MBP) staining was carried out to evaluate the myelin defect after LPS administration. In the LPS-injected group, we found a substantial reduction in the myelin density and auxiliary fiber process in the periventricular white matter compared to the PBS group (Figure 1(b)). LPS is the major type of PAMPs, which stimulates the microglia through Toll-like receptor 4 (TLR-4). Given that microglia contribute to the production and maintenance of the myelin sheath, we wonder whether LPS administration causes damage to the microglial population and destroys their protection of the myelin sheath. To explore the microglial survivability, we isolated those cells from the brain with CD11b^+^ magnetic beads and determined their death rate by quantifying the release of a cell death indicator lactate dehydrogenase (LDH). After culturing ex vivo for 24 hours, LDH release was captured in 40% of microglia from the WMI mice, while in the control mice, only about 10% of microglia released LDH (Figure 1(c)). A bunch of inflammasome activation gives rise to different types of programmed cell death; we then screened the activation of several caspase inflammasomes in the microglia by Fluorochrome Inhibitor of Caspases (FLICA) probe staining. We found that the number of caspase-1-activated microglia doubled in response to LPS-induced WMI, while the activation of other inflammasomes including caspase-3, caspase-6, and caspase-8 stays at the same level in the microglia from the control mice and WMI mice (Figure 1(d)). Caspase-1 activation involves the cleavage of procaspase-1 to an active heterodimer p20 and p10; immunoblot points out an enhanced accumulation of caspase-1 p20 in the microglia from WMI mice compared with controls (Figures 1(e) and 1(f)). Caspase-1 mediated pyroptosis is also identified by the production and secretion of cytokines IL-1*β* and IL-18. We then measured the release of those cytokines by microglia from control and WMI mice with the enzyme-linked immunoassay (ELISA). After 24-hour culture, microglia from control mice released around 200 pg/mL of IL-1*β* and 100 pg/mL of IL-18 into the culture medium (Figures 1(g) and 1(h)). Strikingly, microglia from the WMI mice displayed a robust ability for cytokine production; they produced an average of 380 pg/mL IL-1*β* and 180 pg/mL of IL-18 (Figures 1(g) and 1(h)). Further, dual-color immunofluorescent staining indicates that in the tissue level, there are more Iba1^+^ caspase-1^+^ microglia in the white matter after LPS-induced injury compared with controls (Figure 1(i)). Taken together, these data showed strong activation of inflammasome caspase-1 which induced the pyroptosis of microglia in the WMI mice.

### 2.2. Inactivation of Caspase-1 Attenuates the Pathogenesis of WMI

**Figure 2:**
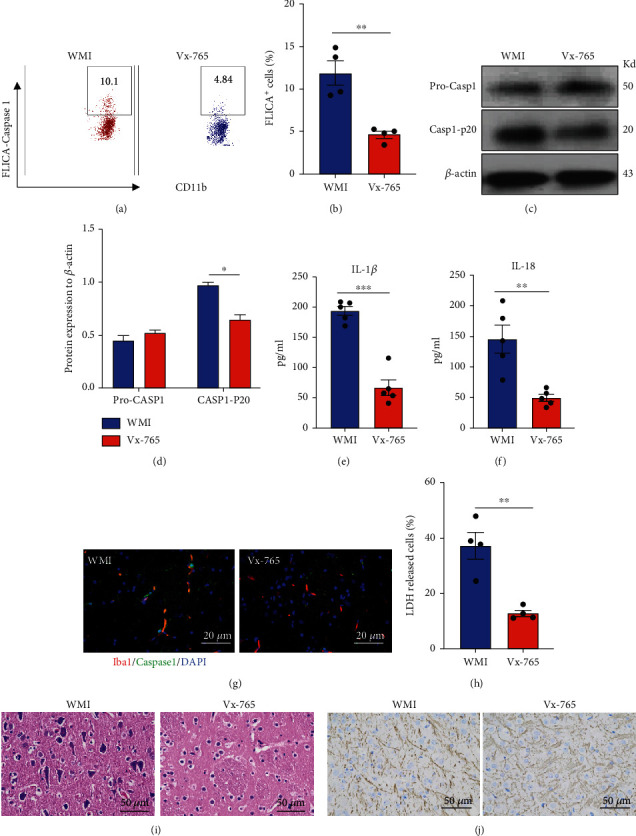
Inactivation of caspase-1 attenuated the pathogenesis of WMI. 25 mg/kg caspase-1 inhibitor Vx-765 injected to the WMI mouse model i.p. Microglia were isolated with a CD11b cell separation kit. (a) FLICA assay staining for caspase 1 (representative dot plot). (b) FLICA assay staining for caspase-1 (statistical data). (c) Immunoblot for procaspase-1 and caspase-1 P20. (d) Relative protein level quantification by normalizing with *β*-actin. (e) IL-1*β* release. (f) IL-18 release. (g) Dual-color staining for lba1 and caspase-1 in the white matter. (h) LDH is released by microglia after ex vivo culture for 24 hours. (i) H&E staining for brain sections. (j) MBP staining for brain sections. Data are mean ± SEM. Individual data points are displayed: ^∗^*P* < 0.05, ^∗∗^*P* < 0.01, and ^∗∗∗^*P* < 0.001.

To investigate whether the caspase-1-dependent pyroptosis of microglia is responsible for white matter injury, we injected caspase-1 inhibitor Vx-765 together with LPS in the WMI induction model. To confirm the inactivation of the caspase-1 inflammasome in microglia, we isolated the microglia from the periventricular white matter after LPS induction. FLICA probe staining shows that Vx-765 successfully reduced the activation of caspase-1 in microglia (Figures 2(a) and 2(b)). Immunoblot further proved the reduction of caspase-1 p20 cleavage after Vx-765 treatment (Figures 2(c) and 2(d)). Further, Vx-765 treatment reduced the production of IL-1*β* and IL-18 by microglia after WMI (Figures 2(e) and 2(f)). Dual-color immunofluorescent staining confirmed the inactivation of caspase-1 in Iba-1^+^ microglia in the white matter after Vx-765 treatment (Figure 2(g)). We then analyze whether blunted caspase-1 activation results in the reverse of WMI. Reduction in LDH production indicates that Vx-765 treatment improves the life span of microglia after WMI (Figure 2(h)). At the tissue level, H&E analysis of brain sections supports that treatment with Vx-765 significantly reduced the lesion area in the white matter after LPS injection (Figure 2(i)). Immunohistochemistry for MBP reveals increased fiber density and myelination in the periventricular white matter area post-Vx-765 injection (Figure 2(j)). Together, these data suggest that pyroptosis of microglia is highly involved in the pathogenic process of WMI. Suppression of caspase-1 activation alleviated the injury of white matter after LPS administration.

### 2.3. Loss of miR-214-3p in Microglia Drives the Pathogenesis of WMI

**Figure 3:**
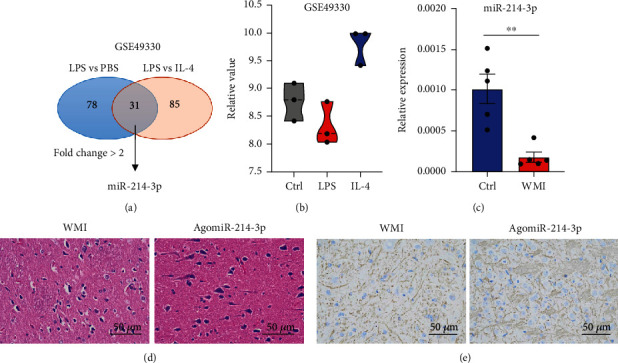
Loss of miR-214-3p in microglia drives the pathogenesis of WMI. (a) Collect data from the GSE49330 database. Different expressed gene numbers between LPS treatment and PBS/IL-4 treatment. Overlapped gene numbers were in red. (b) Relative expression level of miR-214-3p from the GSE49330 database. (c) Relative expression of miR-214-3p in microglia from Ctrl and WMI mice. WMI mice were treated with 40 mg/kg agomiR-214-3p. (d) H&E staining for brain sections. (e) MBP staining for brain sections. Data are mean ± SEM. Individual data points are displayed: ^∗^*P* < 0.01.

MicroRNAs were reported to be highly involved in regulating microglial function from many aspects [18]. To investigate whether there is a functional miRNA that mediates the microglial function in WMI, we pulled out the sequencing data from the GEO database to search for clues. Given that our WMI mouse model was built in a LPS-based manner, we found GSE49330 which identified that the miRNA expression profile on microglia under LPS treatment [19] may be helpful to us. After reanalysis of this database, we found 78 different expressed miRNAs between LPS and PBS treatment. This dataset also allows us to compare the microglial miRNA profile between LPS and IL-4 stimulation; we found 85 different expressed miRNAs when comparing the LPS and IL-4 treatment (Figure 3(a)). The two groups of differently expressed miRNAs have an overlap of 31 miRNAs, of which only one miRNA miR-214-3p exhibits more than 2-fold changes in both groups (Figure 3(a)). In this dataset, LPS treatment dramatically reduced the expression of miR-214-3p on microglia compared with PBS or IL-4 treatment (Figure 3(b)). We then confirmed the expression of miRNA in the microglia of our WMI mice. Strikingly, we found the relative expression of miR-214-3p reduced by more than 3 times in the microglia of WMI mice compared with the control group (Figure 3(c)). To test the function of miR-214 in the progress of WMI, we delivered miR-214 agomir to the WMI mouse model. H&E staining showed that miR-214-3p reduced the white matter lesion area in the LPS-induced mouse model (Figure 3(d)). miR-214-3p agomir also recovered the length and density of myelin in the periventricular white matter (Figure 3(e)). Taken together, the data indicates that the reduction of miR-214-3p expression on microglia leads to the severer pathogenesis of WMI.

### 2.4. miR-214-3p Mediates Caspase-1 Inflammasome Activation in Microglia

**Figure 4:**
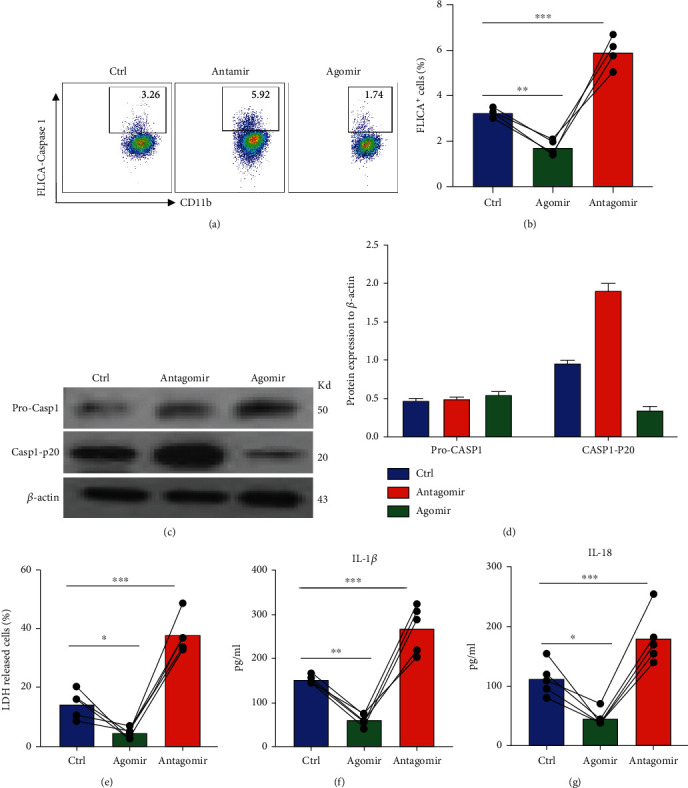
miR-214-3p mediates caspase-1 inflammasome activation in microglia. BV-2 microglia cell lines were cultured and transfected with agomir or antagomir of miR-214-3p. (a) FLICA assay staining for caspase-1 (representative dot plot). (b) FLICA assay staining for caspase-1 (statistical data). (c) Immunoblot for procaspase-1 and caspase-1 P20. (d) Relative protein level quantification by normalizing with *β*-actin. (e) LDH is released by microglia after ex vivo culture for 24 hours. (f) IL-1*β* release in culture medium. (g) IL-18 release in culture medium. Data are mean ± SEM. Individual data points are displayed: ^∗^*P* < 0.05, ^∗∗^*P* < 0.01, and ^∗∗∗^*P* < 0.001.

Next, we tested the role of miR-214-3p in regulating microglial function and inflammasome activation. The microglial cell line BV-2 was transfected with either the agomir or the antagomir of miR-214-3p; caspase-1 activity was first measured by FLICA probe staining. We found that antagomiR-214-3p extensively increased the activity of caspase-1 while the agomiR-214-3p declines the cleavage and activation of the caspase-1 inflammasome (Figures 4(a) and 4(b)). Immunoblot further proved the accumulation of caspase-1 p20 in microglia when miR-214-3p was blocked and the reduction of p20 fragment under agomiR-214-3p transfection (Figures 4(c) and 4(d)). The LDH assay indicates that agomiR214 protects the microglia from cell death while inhibiting miR-214-3p results in a dramatic increase in microglia death (Figure 4(e)). We also determined the production of IL-1*β* and IL-18 after miR-214-3p manipulation. Similarly, agomiR-214-3p prevents BV-22 from producing IL-1*β* and IL-18 while antagomiR-214 promotes the secretion of IL-1*β* and IL-18 by microglia (Figures 4(f)–4(g)). Taken together, these data connect the expression of miR-214-3p to the pyroptosis of microglia.

### 2.5. miR-214-3p Regulates Microglial Pyroptosis in WMI Mice

**Figure 5:**
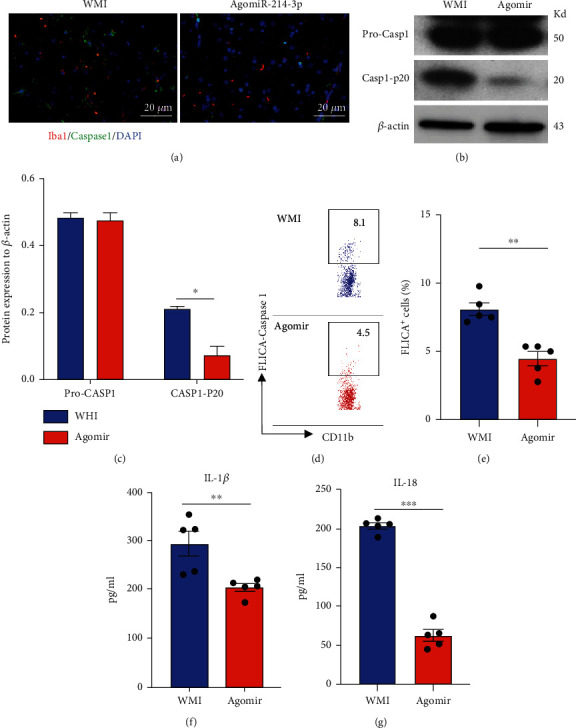
miR-214-3p regulates microglial pyroptosis in WMI mice. 40 mg/kg agomiR-214-3p was injected into the WMI mouse model i.p. Microglia were isolated with a CD11b cell separation kit. (a) Dual-color staining for lba1 and caspase-1 in the white matter. (b) Immunoblot for procaspase-1 P20. (c) Relative protein level quantification by normalizing with *β*-actin. (d) FLICA assay staining for caspase-1 (representative dot plot). (e) FLICA assay staining for caspase-1 (statistical data). (f) IL-1*β* release after 24 hours of culture ex vivo. (g) IL-18 released after 24 hours of culture ex vivo. Data are mean ± SEM. Individual data points are displayed: ^∗^*P* < 0.05, ^∗∗^*P* < 0.01, and ^∗∗∗^*P* < 0.001.

In order to investigate whether miR-214-3p is associated with the caspase-1 activation and microglial pyroptosis in vivo, we injected the agomir of miR-214 while generating the WMI mouse model. Dual-color immunofluorescent staining shows that miR-214-3p suppresses the number of caspase-1^+^Iba^+^ pyroptotic microglia in the white matter after LPS administration (Figure 5(a)). miR-214-3p agomir treatment also reduced the accumulation of caspase-1 p20 protein in the microglia of WMI mice (Figures 5(b) and 5(c)). FLICA probe staining further confirmed that the activation of caspase-1 was blocked by miR-214-3p agomir (Figures 5(d) and 5(e)). The production of IL-1*β* and IL-18 by microglia was suppressed by miR-214 agomir injection (Figures 5(f) and 5(g)). Together, these data indicate that miR-214-3p plays a significant role in regulating the caspase-1-dependent pyroptosis of microglia both in vitro and in vivo.

### 2.6. miR-214-3p Binds to NEK7 to Mediate Caspase-1 Inflammasome Activation in Microglia

**Figure 6:**
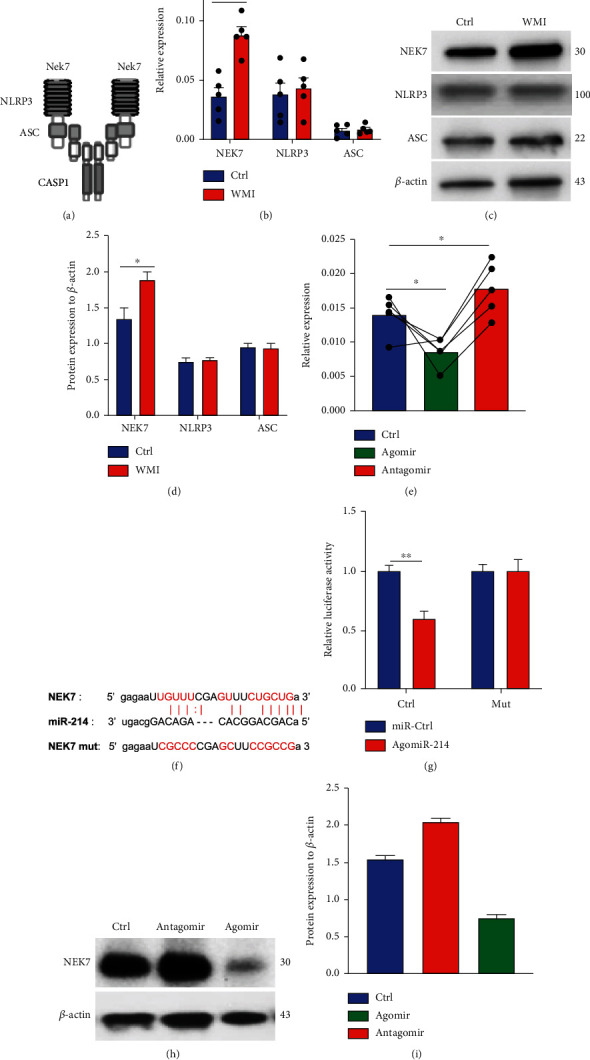
miR-214-3p binds to NEK7 to mediate caspase-1 inflammasome activation in microglia. (a) Scheme graph for the NLRP3 complex. (b) Transcripts of NEK7, NLRP3, and ASC in microglia from Ctrl and WMI mice. (c) Immunoblot for NEK7, NLRP3, and ASC proteins in Ctrl and WMI microglia. (d) Quantification of immunoblot by normalizing with *β*-actin. (e) BV-2 cells were transfected with miR-214-3p agomir and antagomir. NEK7 transcripts were measured by RT-PCR. (f) Assay design for luciferase reporter assay of miR-214 and NEK7 mRNA. (g) Dual-luciferase reporter assay for the binding of miR-214 and NEK7 mRNA. (h) BV-2 cells were transfected with miR-214-3p agomir. NEK proteins were determined by immunoblot. (i) Quantification of NEK7 protein expression after miR-214-39 agomir and antagomir transfection by normalizing with *β*-actin. Data are mean ± SEM. Individual data points are displayed. ^∗^*P* < 0.05, ^∗∗^*P* < 0.01, and ^∗∗∗^*P* < 0.001.

To explore how miR-214-3p regulates the activation of the inflammasome in microglia, we checked the expression of critical components in the NLRP3 complex. Caspase-1 was activated upon the assembly of the inflammasome NLRP3 complex [10]. The NLRP3 complex involves the expression and binding of NEK7, NLRP3, and ASC proteins; each protein is closely associated with the activation of NLRP3 (Figure 6(a)). We first measured the mRNA and protein levels of NLRP3 inflammasome components in microglia from control and WMI mice. An increase in NEK7 mRNA was observed in WMI microglia compared with control cells, while the expression of NLRP3 and ASC transcripts was unchanged before and after WMI induction (Figure 6(b)). At the protein level, the immunoblot shows a clear enhancement of NEK7 expression in microglia from WMI mice compared to controls (Figures 6(c) and 6(d)). No visible difference was found in the NLRP3 and ASC protein levels in the microglia from control and WMI mice (Figures 6(c) and 6(d)). To clarify the role of miR-214-3p in the process of NLRP3 assembly, we treated the BV-2 microglia with either the agomir or the antagomir of miR-214-3p, and the transcription of NEK7, NLRP3, and ASC was measured by RT-PCR. We found that an elevation of miR-214-3p leads to a drop in NEK7 transcripts, while the blockade of miR-214-3p results in the increase in NEK7 transcripts (Figure 6(e)). In the meantime, the transcription of NLRP3 and ASC was not influenced by the variation of miR-214-3p (Figure S1). miRNA regulates gene expression by binding to the 5′ end of mRNA; the starBase database (http://starbase.sysu.edu.cn/) predicts that miR-214-3p is very likely to bind to the mRNA of NEK7 (Figure 6(f)). We then mutated 3′-UTR of NEK7 and performed a luciferase reporter assay to confirm this binding. The relative activity of luciferase proved the binding of miR-214 to the 3′-UTR of NEK7 mRNA (Figure 6(g)). To further confirm this regulation, we treated BV-2 with miR-214-3p agomir and antagomir; the expression of NEK 7 protein was determined by immunoblot. Again, miR-214 antagomir increased the protein level of NEK7 which was reduced by the treatment of miR-214-3p agomir (Figures 6(h) and 6(i)). These data support that miR-214-3p negatively regulates NEK7 expression by binding to the 3′-UTR of its mRNA. Together, this study reported that via suppressing the transcription of NEK7, miR-214-3p reduced the caspase-1-dependent pyroptosis in microglia which attenuates WMI.

## 3. Materials and Methods

### 3.1. Animals

Adult (8-10-week-old) C57BL/6 wide-type mice were purchased from Guangdong Medical Laboratory Animal Center (Fushan, Guangdong, China). The mice were maintained and breed at 12 h light-dark cycle in the specific pathogen-free animal facility. Mice at postnatal day 8 were used for WMI model. Animal experimentation was approved by the Animal Ethics Committee of Guangzhou University of Chinese Medicine (20220114004).

### 3.2. WMI Mouse Model

A postnatal WMI mouse model was created as previously described [20]. In brief, 300 *μ*g/kg lipopolysaccharide (LPS, Sigma-Aldrich) was injected into mice intraperitoneally on postnatal day (PND) 8. After 14 hours, mice were anesthetized with CO_2_; the brain was harvested for further analysis. In some groups, 25 mg/kg caspase-1 inhibitor Vx-765 (Sigma-Aldrich) or 40 mg/kg miR-214 agomir (RiboBio, Guangzhou, China) was injected at the same time.

### 3.3. Microglial Isolation

Mouse brain tissue was disassociated with a tissue homogenizer at low speed for 30 seconds on ice. The homogenized tissue was dissolved with 1.5 mg/mL collagenase IV (Worthington) in a serum-free medium for 45 min at 37°C. Pass the solution through a 40 *μ*m strainer to observe single-cell suspension. Microglia were isolated from the single-cell suspension with a CD11b magnetic positive selection kit following the manufacturer's instruction (Miltenyi Biotec).

### 3.4. Cell Culture

The BV-2 microglia cell line was purchased from AcceGen Biotechnology. BV-2 cells were cultured in DMEM-high glucose media supplemented with 10% fetal bovine serum, 100 IU/mL penicillin, and 100 mg/mL streptomycin.

### 3.5. Cell Transfection

50 nM of agomiR-214-3p or antagomiR-214-3p (RiboBio, Guangzhou, China) was transfected to microglia with the Lipofectamine™ 3000 Transfection Reagent for 24 hours (Thermo Fisher).

### 3.6. Tissue Section and Staining

Mouse brains were harvested and embedded with optimal cutting temperature (OCT) compound and frozen with dry ice. Tissue sections were cut at the thickness of 10 *μ*m. H&E staining was performed with a staining kit following the manufacturer's instruction (Beyotime).

### 3.7. LDH Detection Assay

Microglia from mice were isolated and cultured for 24 hours. The LDH in the supernatant was measured with the LDH Cytotoxicity Assay Kit following the manufacturer's instructions (Thermo Fisher). The percentage of LDH release cells was calculated by dividing total LDH activity by spontaneous LDH release.

### 3.8. FLICA Probe Staining

The FAM-FLICA® Assay Kit for caspase-1, caspase-3, caspase-6, and caspase-8 was purchased from Immunochemistry Technologies. 1 : 30 of FLICA in PBS was incubated with cells for 1 hour at 4°C. Microglia were also labeled with an anti-mouse CD11b antibody. After triple wash, cells were analyzed with a flow cytometer.

### 3.9. Protein Extraction and Western Blot

Cells pellets were lysed with RIPA buffer for 30 min on ice. The proteins were resolved in 4-15% SDS-PAGE (Bio-Rad). Proteins were then transferred to PVDF membranes (Bio-Rad) followed by blockade with 1% BSA in TBST. A caspase-1 antibody (Santa Cruz Biotechnology, 14F468: sc-56036) was diluted to 1 : 1000 with TBST and incubated with the membrane overnight at 4 degrees. The membrane was washed three times with TBST and then incubated with goat anti-mouse IgG-HRP (Santa Cruz Biotechnology, sc-2005, 1 : 5000) for 1 hour at room temperature (https://www.scbt.com/p/goat-anti-mouse-igg-hrp?requestFrom=search). Pierce™ ECL Western Blotting Substrate (Thermo Fisher, 32209) was used to detect protein following the manufacturer's instruction.

### 3.10. ELISA

Microglia were isolated and cultured for 24 hours; the culture supernatant was collected after centrifugation at 350 g for 5 min. IL-1*β* and IL-18 produced by microglia were quantified with the ELISA kit following the manufacturer's instruction (BioLegend).

### 3.11. Immunofluorescence Staining and Imaging

Microglia were labeled with anti-mouse Iba-1 (1 : 100) plus Alexa Fluor® 488 anti-rabbit IgG (1 : 200, Thermo Fisher Scientific). Caspase-1 P20 was detected with anti-mouse caspase-1 (1 : 100) plus Alexa Fluor® 594 anti-mouse IgG (1 : 200, Thermo Fisher Scientific). The image was obtained with a Zeiss Axio Scope A1 microscope (Carl Zeiss, Oberkochen, Germany).

### 3.12. RNA Extraction and Quantitative RT-PCR

Total RNA was extracted using a Trizol RNA isolation kit (Beyotime). Reverse transcription was performed with cDNA Synthesis Kits following the manufacturer's instruction (Thermo Fisher). Quantitative RT-PCR was performed on the Eppendorf Thermal Cycler using the SYBR Green qPCR Master Mix (Bimake). Expression levels were normalized to beta-actin expression and displayed as 2 − ΔCt∗10^−3^.

### 3.13. Dual-Luciferase Reporter Assay

The 3-UTRs of NEK7 and mutant NEK7 were cloned downstream of the firefly luciferase gene in the pGL3 vector (Promega). The reporter assay was performed with the Dual-Luciferase® reporter assay system following the manufacturer's instructions (Promega).

### 3.14. GEO Database Analysis

The significant differentially expressed mRNAs were analyzed with GEO2R140 tools from the R package “limma” in GSE49330 (fold change (FC) > 1.5, *P* < 0.05, and a false discovery rate (FDR) < 0.2).

### 3.15. Statistical Analysis

Data are presented as mean ± standard error of the mean (SEM). *P* < 0.05 was considered statistically significant. Two-tailed unpaired Student's *t*-test and one-way ANOVA were used to compare groups. All statistical analyses were performed by using GraphPad Prism 8.0 (GraphPad Software Inc.).

## 4. Discussion

White matter injury (WMI) is one of the most severe pathological processes in premature infants and newborns that affect the development of their central nervous system [21]. In the past few years, studies have suggested that the immune cell microglia appear to play an essential role during the pathogenesis of WMI [6, 22, 23]. However, how microglia regulate WMI development and the mechanism underlying it was not well understood. In this study, we found an increase in caspase-1 inflammasome-dependent pyroptosis in the microglia of WMI mice compared with normal controls. Blockade of caspase-1 activity is able to reduce the white matter lesion area and reverse the dysmyelination after WMI. The activation of caspase-1 inflammasome is regulated by the level of microRNA miR-214 in the microglia. The expression of miR-214-3p dramatically dropped in the microglia of WMI mice. Induction of miR-214-3p prevents the pyroptosis of BV-2 microglia while inhibition of this miRNA results in an increase in caspase-1 activation and pyroptosis of BV-2 microglia. Administration of miR-214-3p agomir to the WMI model successfully alleviates the degree of white matter damage and loss of myelination; this suggests the involvement of miR-214-3p in the pathogenesis of WMI. Mechanistically, miR-214-3p binds to the 3′ region on NEK7 mRNA, which is a significant component of the NLRP3 complex supporting the activation of the NLRP3 inflammasome. Our data delineate a possible therapeutic target for WMI patients to relieve their pathologic lesions and increase their life quality.

During myelin development, microglia in the white matter are much more active than those cells in the grey matter [24]. This implies that the activation and heterogeneity of microglia are highly involved in the axon demyelination and lesion formation of premature newborn WMI [21, 25]. Under normal conditions, microglia express certain markers including CD11c, Mac2, Clec7a, and Spp1 [26]. By expressing growth factors like IGF-1, the role of microglia is to protect and support myelin build-up [27]. During damage, microglia first turned to a proinflammatory phenotype that expresses CD68, MHCI/II, CR3, and iNOS; these microglia will induce the axon demyelination and lesion formation [6]. The inflammatory status was also closely related to diseases in the central nerve system [27]. Comprehensive analysis of cerebrospinal fluid demonstrates a highly distinctive inflammation environment in preterm infants; hallmarks of proinflammatory units including IL-1*β*, IL-9, TNF-*α*, and complement component 5a (C5a) are highly expressed [28, 29].

Several different animal models of WMI were established, of which administration of LPS or induction of hypoxia-ischemia gives rise to the most notable outcome [30, 31]. Besides enhanced proinflammatory action, hypomyelination, and oligodendrocyte immigration, overall activation of the NLRP3 inflammasome was also noticed in these animal models [32, 33]. Increased formation of IL-1*β* and IL-18 was reported in the neonatal hypoxic-ischemic injury [34]. In this current study, we reported that after LPS injection, the activation of caspase-1 inflammasome increased in the microglia which is responsible for the expansion of white matter lesion and myelin reduction. Thus, treating WMI by manipulating the cleavage and activation of caspase-1 deserves to be considered.

In recent decades, various miRNAs were reported to participate in different stages of brain development as well as injury pathogenesis. As a major part of the white matter, the development and damage of oligodendrocytes appear to be regulated by miR-219 [35]. miR-338 and miR-138 are the other key regulators of oligodendrocytes especially under hypoxia-ischemia condition [16]. An important miRNA for microglia polarization and activation is miR-124-3p; overexpression of miR-214-3p induces a quiescent state of microglia by targeting the CEBP*α*/PU.1 signal [36]. miR-155 and miR-27a were reported to regulate the inflammation states and nitric oxide production of microglia [37, 38]. Here, our data demonstrated that through binding to NEK7, miR-214-3p regulates caspase-1 inflammasome activation and microglial pyroptosis induction. Further studies will be necessary to evaluate the potential of miR-214-3p as a therapeutic target for WMI patients.

## Data Availability

All data in the manuscript are available. The corresponding author can provide detailed raw data when necessary.

## Supplementary Materials

Supplementary Materials Overactivation of caspase-1 inflammasome causes increased pyroptosis of microglia in white matter injury (WMI). Pharmacology blockade of inflammasome activation rescues the pathogenesis of WMI mice. The reduced miR-214-3p expression on microglia suppresses the transcription of NEK7, the NLRP-3 inflammasome compartment mRNA.

## References

[1] Hagberg H., Mallard C., Ferriero D. M. (2015). The role of inflammation in perinatal brain injury. *Nature Reviews. Neurology*.

[2] Martinez-Biarge M., Groenendaal F., Kersbergen K. J. (2019). Neurodevelopmental outcomes in preterm infants with white matter injury using a new MRI classification. *Neonatology*.

[3] Gefen T., Kim G., Bolbolan K. (2019). Activated microglia in cortical white matter across cognitive aging trajectories. *Frontiers in Aging Neuroscience*.

[4] Wang Q., Yang W., Zhang J., Zhao Y., Xu Y. (2020). TREM2 overexpression attenuates cognitive deficits in experimental models of vascular dementia. *Neural Plasticity*.

[5] Xu Y., Wang Q., Wu Z. (2019). The effect of lithium chloride on the attenuation of cognitive impairment in experimental hypoglycemic rats. *Brain Research Bulletin*.

[6] McNamara N. B., Miron V. E. (2020). Microglia in developing white matter and perinatal brain injury. *Neuroscience Letters*.

[7] Zhang L. Y., Pan J., Mamtilahun M. (2020). Microglia exacerbate white matter injury via complement C3/C3aR pathway after hypoperfusion. *Theranostics*.

[8] Saijo K., Crotti A., Glass C. K. (2013). Regulation of microglia activation and deactivation by nuclear receptors. *Glia*.

[9] Kelley N., Jeltema D., Duan Y., He Y. (2019). The NLRP3 inflammasome: an overview of mechanisms of activation and regulation. *International Journal of Molecular Sciences*.

[10] Swanson K. V., Deng M., Ting J. P. (2019). The NLRP3 inflammasome: molecular activation and regulation to therapeutics. *Nature Reviews. Immunology*.

[11] He Y., Zeng M. Y., Yang D., Motro B., Nunez G. (2016). NEK7 is an essential mediator of NLRP3 activation downstream of potassium efflux. *Nature*.

[12] Du X., Xu Y., Chen S., Fang M. (2020). Inhibited CSF1R alleviates ischemia injury via inhibition of microglia M1 polarization and NLRP3 pathway. *Neural Plasticity*.

[13] Ran Y., Su W., Gao F. (2021). Curcumin ameliorates white matter injury after ischemic stroke by inhibiting microglia/macrophage pyroptosis through NF-*κ*B suppression and NLRP3 inflammasome inhibition. *Oxidative Medicine and Cellular Longevity*.

[14] Macfarlane L. A., Murphy P. R. (2010). MicroRNA: biogenesis, function and role in cancer. *Current Genomics*.

[15] Zamani P., Oskuee R. K., Atkin S. L., Navashenaq J. G., Sahebkar A. (2020). MicroRNAs as important regulators of the NLRP3 inflammasome. *Progress in Biophysics and Molecular Biology*.

[16] Xiao D., Qu Y., Pan L., Li X., Mu D. (2018). MicroRNAs participate in the regulation of oligodendrocytes development in white matter injury. *Reviews in the Neurosciences*.

[17] Ahmad A. S., Satriotomo I., Fazal J., Nadeau S. E., Dore S. (2015). Considerations for the optimization of induced white matter injury preclinical models. *Frontiers in Neurology*.

[18] Lemaire Q., Raffo-Romero A., Arab T. (2019). Isolation of microglia-derived extracellular vesicles: towards miRNA signatures and neuroprotection. *Journal of Nanobiotechnology*.

[19] Freilich R. W., Woodbury M. E., Ikezu T. (2013). Integrated expression profiles of mRNA and miRNA in polarized primary murine microglia. *PLoS One*.

[20] Wang X., Stridh L., Li W. (2009). Lipopolysaccharide sensitizes neonatal hypoxic-ischemic brain injury in a MyD88-dependent manner. *Journal of Immunology*.

[21] Back S. A. (2017). White matter injury in the preterm infant: pathology and mechanisms. *Acta Neuropathologica*.

[22] Lee J., Hamanaka G., Lo E. H., Arai K. (2019). Heterogeneity of microglia and their differential roles in white matter pathology. *CNS Neuroscience & Therapeutics*.

[23] Shao R., Sun D., Hu Y., Cui D. (2021). White matter injury in the neonatal hypoxic-ischemic brain and potential therapies targeting microglia. *Journal of Neuroscience Research*.

[24] Hughes A. N., Appel B. (2020). Microglia phagocytose myelin sheaths to modify developmental myelination. *Nature Neuroscience*.

[25] Back S. A. (2014). Cerebral white and gray matter injury in newborns: new insights into pathophysiology and management. *Clinics in Perinatology*.

[26] Benmamar-Badel A., Owens T., Wlodarczyk A. (2020). Protective microglial subset in development, aging, and disease: lessons from transcriptomic studies. *Frontiers in Immunology*.

[27] Wang X., Wang Q., Wang K. (2022). Is immune suppression involved in the ischemic stroke? A study based on computational biology. *Frontiers in Aging Neuroscience*.

[28] Boardman J. P., Ireland G., Sullivan G. (2018). The cerebrospinal fluid inflammatory response to preterm birth. *Frontiers in Physiology*.

[29] Hellström Erkenstam N., Smith P. L., Fleiss B. (2016). Temporal characterization of microglia/macrophage phenotypes in a mouse model of neonatal hypoxic-ischemic brain injury. *Frontiers in Cellular Neuroscience*.

[30] Huang Z., Liu J., Cheung P. Y., Chen C. (2009). Long-term cognitive impairment and myelination deficiency in a rat model of perinatal hypoxic-ischemic brain injury. *Brain Research*.

[31] Nobuta H., Ghiani C. A., Paez P. M. (2012). STAT3-mediated astrogliosis protects myelin development in neonatal brain injury. *Annals of Neurology*.

[32] Verney C., Monier A., Fallet-Bianco C., Gressens P. (2010). Early microglial colonization of the human forebrain and possible involvement in periventricular white-matter injury of preterm infants. *Journal of Anatomy*.

[33] Serdar M., Kempe K., Rizazad M. (2019). Early pro-inflammatory microglia activation after inflammation-sensitized hypoxic-ischemic brain injury in neonatal rats. *Frontiers in Cellular Neuroscience*.

[34] Girard S., Sebire G., Kadhim H. (2010). Proinflammatory orientation of the interleukin 1 system and downstream induction of matrix metalloproteinase 9 in the pathophysiology of human perinatal white matter damage. *Journal of Neuropathology and Experimental Neurology*.

[35] Dugas J. C., Cuellar T. L., Scholze A. (2010). Dicer1 and miR-219 are required for normal oligodendrocyte differentiation and myelination. *Neuron*.

[36] Ponomarev E. D., Veremeyko T., Barteneva N., Krichevsky A. M., Weiner H. L. (2011). MicroRNA-124 promotes microglia quiescence and suppresses EAE by deactivating macrophages via the C/EBP-*α*-PU.1 pathway. *Nature Medicine*.

[37] Lv Y. N., Ou-Yang A. J., Fu L. S. (2017). MicroRNA-27a negatively modulates the inflammatory response in lipopolysaccharide-stimulated microglia by targeting TLR4 and IRAK4. *Cellular and Molecular Neurobiology*.

[38] Cardoso A. L., Guedes J. R., Pereira de Almeida L., Pedroso de Lima M. C. (2012). miR-155 modulates microglia-mediated immune response by down-regulating SOCS-1 and promoting cytokine and nitric oxide production. *Immunology*.

