# Corrigendum to “miR-214-3p Deficiency Enhances Caspase-1-Dependent Pyroptosis of Microglia in White Matter Injury”

**DOI:** 10.1155/jimr/9826132

**Published:** 2025-08-13

L. He, T. Wei, Y. Huang, et al., “miR-214-3p Deficiency Enhances Caspase-1-Dependent Pyroptosis of Microglia in White Matter Injury”, *Journal of Immunology Research* 2022 (2022): 1642896, https://doi.org/10.1155/2022/1642896.

In the article, it is incorrectly stated that mice were anesthetized after 14 h. The authors have clarified that the mice were anesthetized after 24 h.

This error does not affect the conclusions of the article.

We apologize for this error.

