# Supplementary material for: miR-214-3p Deficiency Enhances Caspase-1-Dependent Pyroptosis of Microglia in White Matter Injury
Source: J Immunol Res. 2022 Aug 22;2022:1642896. doi: 10.1155/2022/1642896 (PMC11390193; doi:10.1155/2022/1642896)
Supplement: Supplementary Materials — Overactivation of caspase-1 inflammasome causes increased pyroptosis of microglia in white matter injury (WMI). Pharmacology blockade of inflammasome activation rescues the pathogenesis of WMI mice. The reduced miR-214-3p expression on microglia suppresses the transcription of NEK7, the NLRP-3 inflammasome compartment mRNA. [file 1642896.f1.pdf]

Fig S1.

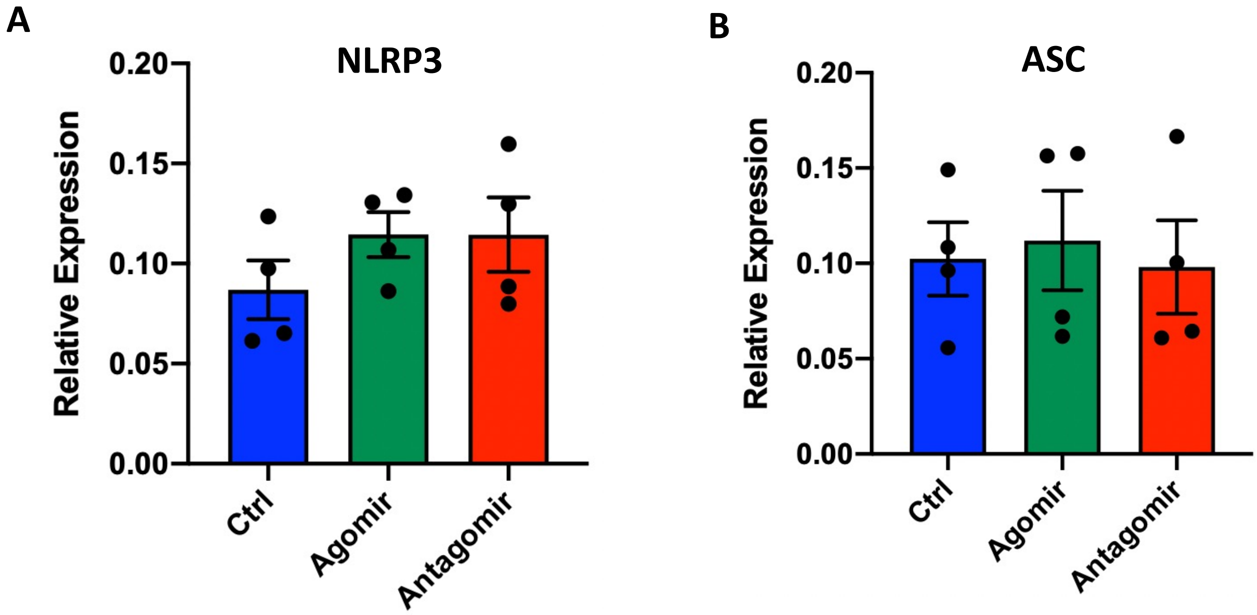

Fig S1.

BV-2 cells were transfected with miR-214 Agomir and Antagomir.

A. NLPR3 transcripts were measured by RT-PCR.

B. ASC transcripts were measured by RT-PCR.
